# Predicting non-melanoma skin cancer via a multi-parameterized artificial neural network

**DOI:** 10.1038/s41598-018-19907-9

**Published:** 2018-01-26

**Authors:** David Roffman, Gregory Hart, Michael Girardi, Christine J. Ko, Jun Deng

**Affiliations:** 1 Department of Therapeutic Radiology, School of Medicine, Yale University, New Haven, USA; 2 Department of Dermatology, School of Medicine, Yale University, New Haven, USA

## Abstract

Ultraviolet radiation (UVR) exposure and family history are major associated risk factors for the development of non-melanoma skin cancer (NMSC). The objective of this study was to develop and validate a multi-parameterized artificial neural network based on available personal health information for early detection of NMSC with high sensitivity and specificity, even in the absence of known UVR exposure and family history. The 1997–2015 NHIS adult survey data used to train and validate our neural network (NN) comprised of 2,056 NMSC and 460,574 non-cancer cases. We extracted 13 parameters for our NN: gender, age, BMI, diabetic status, smoking status, emphysema, asthma, race, Hispanic ethnicity, hypertension, heart diseases, vigorous exercise habits, and history of stroke. This study yielded an area under the ROC curve of 0.81 and 0.81 for training and validation, respectively. Our results (training sensitivity 88.5% and specificity 62.2%, validation sensitivity 86.2% and specificity 62.7%) were comparable to a previous study of basal and squamous cell carcinoma prediction that also included UVR exposure and family history information. These results indicate that our NN is robust enough to make predictions, suggesting that we have identified novel associations and potential predictive parameters of NMSC.

## Introduction

Skin cancer is the most commonly occurring malignancy, with basal and squamous cell carcinomas (both of which are classified as non-melanoma) comprising the majority of skin cancer cases^1^. As non-melanoma skin cancer (NMSC) is often not reported to cancer registries, there is a plethora of literature which attempts to estimate the number of new cases each year. A study cited on the American Cancer Society’s website indicates that 3.3 million people each year will be diagnosed, with about 80% of those cases being basal cell carcinoma (BCC)^1^. Other studies that rely on Medicare and Medicaid statistics have estimated the number of Americans in 2012 with NMSC was as high as 5.4 million with 3.3 million people being treated that year^2^. This is a substantial increase when compared to a 2006 study by members of the same group, that estimated 3.5 million Americans developed NMSC with a resulting 2.1 million procedures performed that year^3^. Another study in Europe identified the incidence of NMSC at 0.145% for men and 0.119% for women^4^ with the annual NMSC cases in Germany estimated at 120,000 and 200,000 per year respectively.

BCC is typically slow growing and almost always localized to the skin, with a metastatic rate of less than 0.1%^5^. Cutaneous SCC, in contrast, has an overall metastatic rate of 0.3–3.7%, with higher rates for certain locations, e.g. ear and lip^6^. Two of the major established risk factors for NMSC are a personal history of chronic ultraviolet radiation (UVR) exposure and a family history of NMSC^7^. Lifestyle choices can affect risk, including a history of smoking and the use of tanning salons that can increase the risk of SCC by a factor of 2.3^8^ and 2.5^9^, respectively.

Recently there has been an interesting study reporting a dermatologist-level classification of skin cancer via a single convolutional neural network trained with a dataset of 129,450 clinical images^10^. This study exemplifies the potential of harnessing available large datasets for efficient and practical use in clinical dermatology. Instead of skin cancer classification based on images, the question we seek to address herein is: can an artificial neural network (ANN) trained with a large set of health informatics that lacks any UVR exposure and family history of NMSC information be used to predict personal NMSC risk, and if so how well would such a network perform? Our approach is novel and significant as it requires only personal health informatics commonly available in the electronic medical record (EMR) systems, and is therefore a convenient and cost-effective method of evaluating cancer risk for individuals. The parameters used in our model are gender^11^, age^12^, body mass index (BMI)^13^, diabetic status^14,15^, smoking status^16^, emphysema, asthma^17^, race^18^, Hispanic ethnicity, hypertension, heart diseases, vigorous exercise habits^19^, and history of stroke. While not all of these parameters are known to be associated with NMSC, we include them because they are readily available information and the nonlinearity of our ANN means that they can have a bigger impact on our accuracy than traditional statistical methods predict. Our aim was achieved with an area under the curve (AUC) of 0.8 or above.

## Materials and Methods

### Data sets

The National Health Interview Survey (NHIS) adult files are surveys that are publicly available^20^ with the corresponding manuals and criteria included (which vary by year). We used the NHIS datasets from 1997–2015 with the exception of 2004 due to a problem in the NHIS data file. The 2015 set has a direct csv format viewing option, with all the other years having SAS code provided to assist the user in extracting the data. The response rate for NHIS adult survey is approximately 80%^21^ and we are only able to view the data that has been collected and filtered by the NHIS.

As only the 2005, 2010, and 2015 survey years contain family history of cancer (adult cancer files) and complete answers are required for our ANN, we did not use these family history files in this study. The demographics of the sample used are shown in Table 1. Notably, the NHIS records all people over the age of 85 as age 85, and these people were included in our training and validation sets.

**Table 1:** The demographics of the NHIS dataset that was used in our ANN.

Demographics of the Data	NMSC Cancer	Non-Cancer
Average Age	62.37 [61.81, 62.93]	46.12 [46.02, 46.22]
Average BMI	27.05 [26.80, 27.30]	27.30 [27.28, 27.32]
Male/Female	47.89%/52.11%	45.11%/54.89%
Ever Smoked	49.64% [47.32%, 52.00%]	41.95% [41.78%, 42.12%]
Have Emphysema	3.710% [2.82%, 4.59%]	1.527% [1.48%, 1.57%]
Have Asthma	12.21% [10.67%, 13.73%]	11.04% [10.93%, 11.15%]
Have Diabetes Mellitus	11.49% [10.02%, 13.01%]	7.826% [7.73%, 7.92%]
Have Ever Had a Stroke	4.828% [3.84%, 5.85%]	2.507% [2.45%, 2.56%]
Have Hypertension	47.45% [45.10%, 49.77%]	27.38% [27.23%, 27.53%]
Average Heart Disease Score	0.097 [0.0877, 0.106]	0.040 [0.0395, 0.0405]
White	97.89% [97.22%, 98.56%]	77.23% [77.09%, 77.37%]
African-American	0.479% [0.14%, 0.77%]	15.48% [15.36%, 15.60%]
Native American/Alaska Native	0.040% [0%, 0.17%]	0.851% [0.82%, 0.88%]
Asian	0.599% [0.26%, 1.00%]	4.925% [4.85%, 5.00%]
Multiracial	0.998% [0.26%, 1.00%]	1.514% [1.47%, 1.56%]
Hispanic Ethnicity	1.756% [1.15%, 2.38%]	16.96% [16.83%, 17.09%]
Average Number of Times Vigorous Exercise is done at Least Once per Week	1.511 [1.388, 1.634]	1.597 [1.587, 1.607]

95% confidence intervals are shown in brackets. Percentages are Wald statistic^24^ and raw numbers are Z statistic.

We used 70% of the data (1,754 NMSC cases and 322,402 never cancer cases) for training and 30% for validation (752 NMSC cases and 138,172 never cancer cases). These data sets were chosen randomly without replacement from the full data set with the restriction that the proportion of cancer and non-cancer cases was kept constant. To be included in our ANN, the first cancer had to be NMSC cancer and occur within 4 years of the survey date. Several of the inputs for our ANN are time-dependent, such as BMI and diabetic status. We selected a four year cutoff as a compromise between the time-dependent aspects of the problem and the sample size restriction required for training. Note that this four year cutoff only applies to the cancer group. However, after testing different cutoff values, it was revealed that they had little impact on the results. Essentially we selected this four year cutoff to strike a balance between sufficient sample size and robust predicting power.

### Implementation of an artificial neural network (ANN)

**Figure 1:**
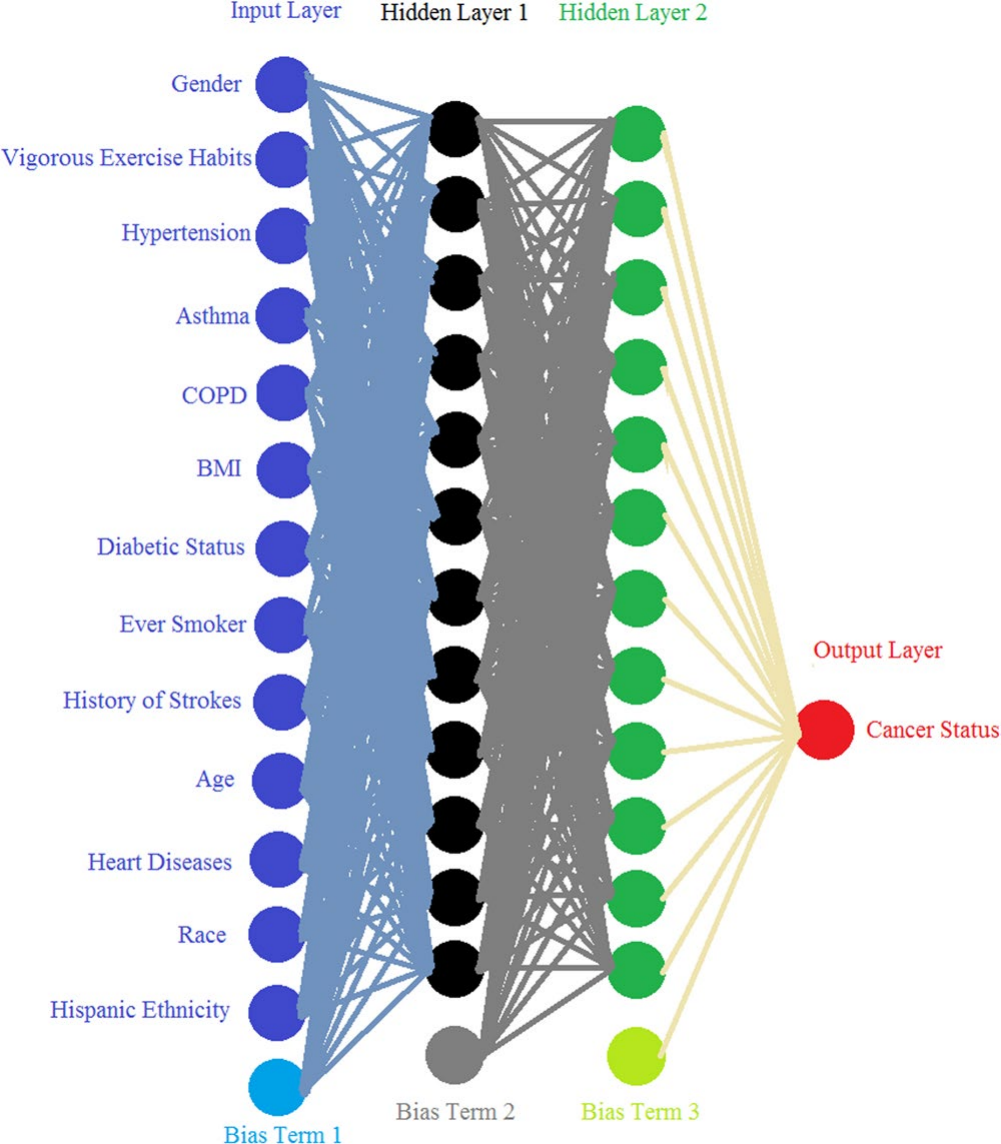
A Schematic of the ANN. Each line is weight connecting one layer to next, with each circle representing an input, neuron, or output. The bias terms are analogous to intercepts and improve the model’s performance.

For this ANN model, we used 12 neurons in each layer and both genders were considered. A schematic of our ANN is shown in Fig. 1. As is standard, our ANN relies on a backpropagation algorithm with bias terms that uses gradient descent^22^ taking the whole training dataset at once. Inputs were normalized to fall in between 0 and 1 and the activation function was sigmoid. A modification was made to this algorithm to allow further speedup of convergence by increasing the learning rate each time the cost function decreases and decreasing the learning rate while resetting the weights to the last iteration if the cost function increases, similar to the momentum approach^23^.

Listed in Table 2 are all the personal health parameters modeled in the ANN. Some parameters are rescaled to comply with the mathematical format required in ANN while others take binary inputs.

**Table 2:** A description of the personal health parameters used in the ANN.

Parameter	Input Type	Input Range	Details
Age	Continuous	0.2118–1	Age range is 18–85, with 85+ being treated as 85.
BMI	Continuous	0–1	BMI above 99.95 is treated as 99.95.
Ever Smoker	Binary	0 or 1	Never-smokers are 0 and current and former smokers are 1.
Emphysema	Binary	0 or 1	No COPD is 0 and COPD is 1.
Asthma	Binary	0 or 1	No asthma is 0 and asthma is 1.
Diabetic Status	Binary	0 or 1	Non-diabetics and pre-diabetics are 0, with diabetics being 1.
Strokes	Binary	0 or 1	No strokes is 0 and having a prior stroke is 1.
Hypertension	Binary	0 or 1	No recording of hypertension is 0, and having single measurement of it is 1.
Heart Disease Score	Continuous	0–1	Coronary heart disease, angina, heart attacks, and other heart complications each contribute 0.25 to the score.
Race	Continuous	0.0083–1	Each race is assigned a value equal to its fractional percentage in the sample plus the fractional percentage of each less common race being added to the race of interest.
Hispanic Ethnicity	Binary	0 or 1	No Hispanic ethnicity is 0 and having Hispanic ethnicity is 1.
Vigorous Exercise	Continuous	0–1	Number of times per week vigorous exercise is performed, with 28+ being treated as 28. All years criteria was 20 minutes or more, with the exception of the 2015 which was 10 minutes.
Gender	Binary	0 or 1	0 is a man and 1 is a woman.

With personal health information as the input, the output of our ANN is a fractional number between 0 and 1, with higher values meaning higher cancer risk. To convert the fractional cancer risk value into a binary cancer status (Yes or No) as shown in Fig. 1, a cutoff is introduced above which our ANN would predict a Yes cancer status. Once the training is complete, the algorithm then tests a variety of cutoff values to allow for the computation of sensitivity and specificity. With the cutoff value selected from the training set, that same value is then used on the validation set and the same 2 quantities (sensitivity, specificity) are computed.

### Data Availability

All data used in this paper is publicly available through the CDC’s website. At the time of submission, the url: https://www.cdc.gov/nchs/nhis/data-questionnaires-documentation.htm, goes directly to the webpage from which each year of NHIS data can be found.

## Results

### Sensitivity, specificity, and AUC of our ANN

For the training set, the sensitivity was 88.5% (95% Wald CI^24^, 87.0–90.0%) and specificity was 62.2% (95% Wald CI^24^, 62.1–62.4%) for the prediction of NMSC. The validation set showed a comparable sensitivity of 86.2% (95% Wald CI^24^, 83.7–88.6%) and specificity of 62.7% (95% Wald CI^24^, 62.4–62.9%).

**Figure 2:**
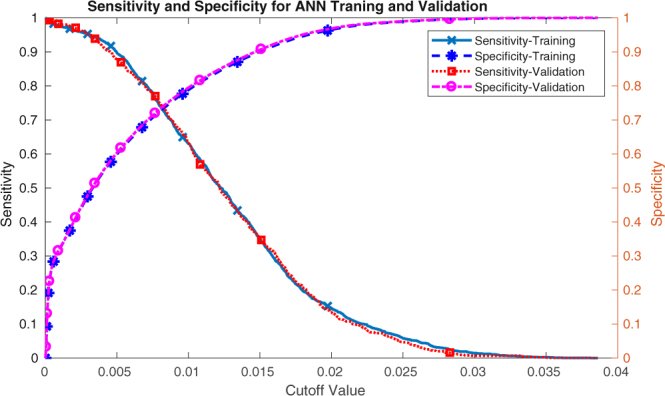
The sensitivity and specificity for the training and validation datasets as functions of the cutoff values.

Since the program computes both quantities of interest for both the training and validations sets, it is important to show how they vary as function of the cutoff values. These results are shown in Fig. 2.

**Figure 3:**
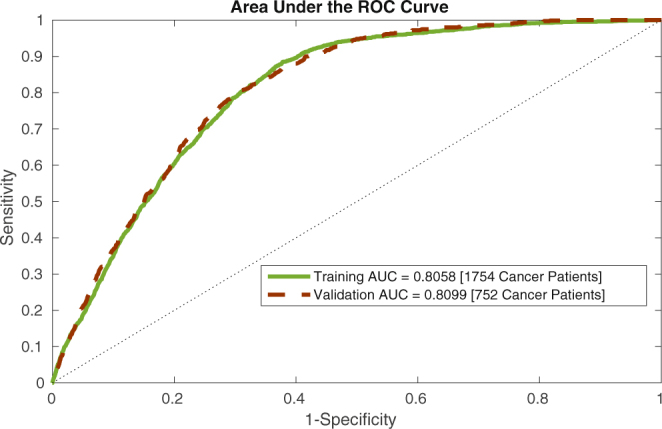
An ROC plot for our ANN’s training and validation datasets.

This information is also conveyed though a conventional receiver operating characteristic (ROC) plot for both the training and validation sets in Fig. 3. Our training and validation sets yielded AUC values of 0.81 (95% CI 0.80–0.82) and 0.81 (95% CI 0.79–0.82), respectively.

### Analysis of NHIS 2016 dataset

**Figure 4:**
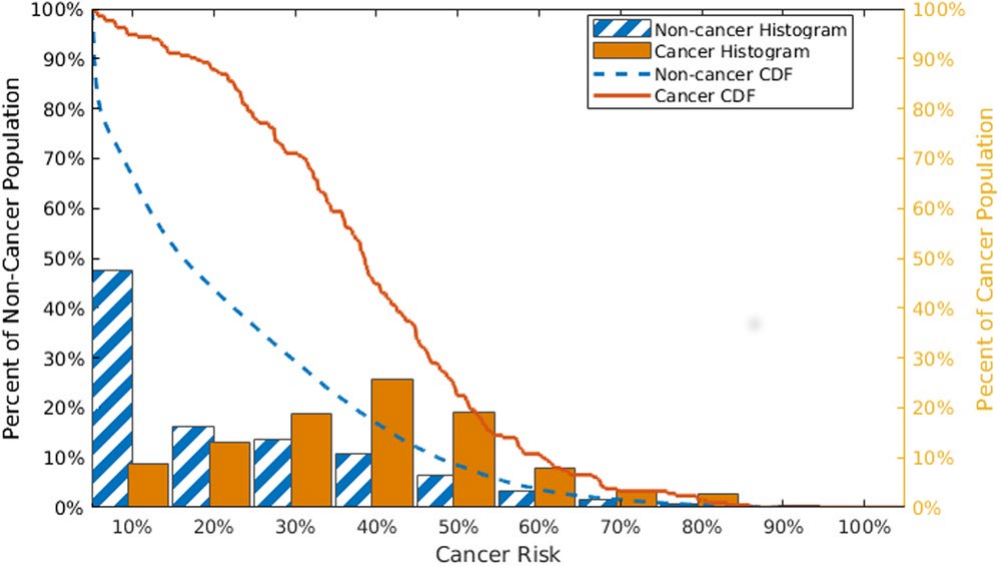
The non-cancerous (blue and white strip/dash) and cancerous (solid orange) people in each risk bin (histograms) and the cumulative distribution functions above a certain risk level (lines).

We have further tested the utility of our model by examining the NHIS 2016 survey data, running the 28,058 individuals from this data set through our ANN. Rather than applying a cutoff and getting a binary answer, we keep the continuous output from our model and normalize it based on the maximum output from the training and validation sets. This transforms the output to between 0 and 1 to predict the percent cancer risk. We examine this risk prediction in Fig. 4 where the percentage of people with cancer and without cancer vary at different cancer risk levels. We also display something akin to an inverse cumulative distribution function (CDF) where the percentage for people with and without cancer at or above a certain cancer risk are shown. While the distribution of prediction risk for cancerous and non-cancerous people overlap, the centers of the histogram distributions are well separated. Also there is a significant gap between the CDFs. These results show that our ANN, drawing upon the most basic of health information, is capable of stratifying people into different cancer risk categories, allowing us to determine who should receive a more thorough and stringent screening for NMSC. As discussed below, these predictions would expect to be substantially improved by adding additional information (UVR exposure and family history) when available.

### A risk stratification tool

**Figure 5:**
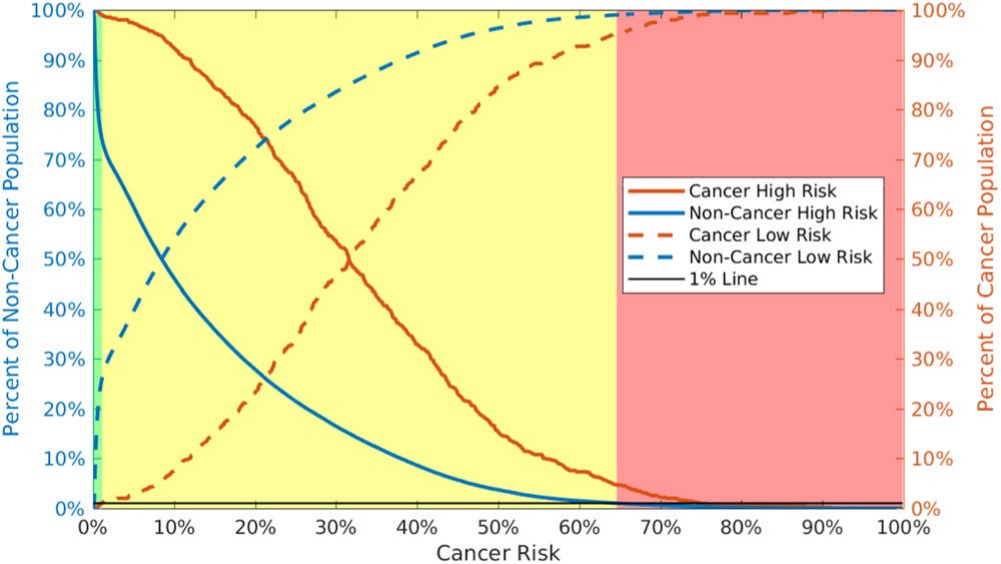
Cancerous (orange) and non-cancerous (blue) people have very different high (solid) and low (dashed) risk trends. Assuming a 1% miss classification rate in the low and high risk categories (black line), we can divide individual cancer risk into 3 categories shown by the shading: high (red), medium (yellow), and low (green).

We take this risk calculation a step further, by translating it into a simple risk stratification tool. As shown in Fig. 5, we select two risk boundaries that break the risk into 3 categories: high risk (represented by red), medium risk (yellow), and low risk (green). In this scheme high risk people should be screened immediately, while medium risk people should receive their standard regular screenings, and low risk people could be screened less frequently. We chose the boundary between medium and high risk (64.6% risk) so that only 1% of the non-cancerous individuals would be classified as high risk. Likewise, the boundary between low and medium risk (1.09%) was chosen such that only 1% of the cancerous individuals would be classified as low risk. Notably, our choices for the two boundaries are conservative. If the cost and risk in screening non-cancerous patients is low, we may move the medium-high risk boundary to capture more cancerous patients in the high risk category. We tested the stratification scheme on the 2016 NHIS data which was not included in either our training or validation data sets. At the boundaries we set we eliminate ~20% of non-cancerous individuals from regular screenings and flag almost 4% of the cancerous individuals for immediate screening (see Table 3). We have further tested our tool using the NHIS datasets of 1997 to 2015. Because of data variation in each individual year, our tool performed better in terms of sensitivity and specificity in some years (e.g., 2007) and less well in some other years (e.g., 2008). Overall, our tool performed well on each year’s NHIS dataset with about 0–3% of the cancerous population in low risk zone, 2–8% in high risk zone, and 90–97% in the medium risk zone.

**Table 3:** Comparison of risk stratification results between NHIS 1997–2015 data and NHIS 2016 data.

		# Respondents	# Low Risk	% Low Risk	# Medium Risk	% Medium Risk	# High Risk	% High Risk
Training 1997–2015	Cancer	1,754	24	1.37%	1625	92.7%	105	6.00%
	Non-Cancer	32,2402	83,279	25.8%	235,542	73.1%	3,581	1.11%
Validation 1997–2015	Cancer	752	8	1.06%	709	94.3%	35	4.65%
	Non-Cancer	138,172	35,976	26.0%	100,813	73.0%	1,381	1.00%
2016	Cancer	214	3	1.40%	203	94.9%	8	3.74%
	Non-Cancer	27,844	5,643	20.3%	21,753	78.1%	448	1.61%

## Discussion

In this study, we presented a multi-parameterized artificial neural network that can be used to predict and stratify NMSC risk based solely on personal health data. Our results are clinically compelling for several reasons. Firstly, our ANN was trained and validated on the NHIS 1997–2015 big data sets with 463,080 respondents, and further tested with the NHIS 2016 data set of 28,058 respondents. Secondly, the developed ANN was able to assess NMSC risk with a high sensitivity while having a decent specificity, comparable to most of the current approaches which often require ultraviolet radiation exposure and family history data. Yet, our model only requires personal health data which is readily available in the EMR system. Thirdly, using simple and easily obtainable personal health data we can stratify patients by cancer risk into three categories: high, medium, and low, which could offer clinical decision support and individualized cancer risk management. As such, our model is a predictive one that can help focus diagnostic resources on the most vulnerable patients. With the addition of additional parameters such as ultraviolet radiation exposure^7^ and family history of NMSC^7^, as well as characteristics of the potential lesions, our model could potentially become a diagnostic tool in addition to a predictive one. We are currently investigating how to include family history data from the NHIS years for which it is available.

Our current approach is distinguished from a previous study that utilized logistic regression for NMSC diagnosis by analyzing survey data from a much smaller sample size, and that also contained radiation exposure (e.g. sun exposure, sun burn history, tanning salon usage,) and family history^25^. This survey-based analysis was limited to ~200 adults who were referred by their primary physicians for suspicious skin lesions, e.g. to rule out actinic keratosis or NMSC. The patient’s questionnaires were filled in by the nurses who also recorded some of the characteristics of the lesions. Depending on the model used and type of NMSC attempting to be diagnosed, the AUC scores varied in between 0.78–0.93. Our ANN yields an AUC score that is similar to their worst model, but pales in comparison to their better models. This is expected as UVR exposure information and family history play such major roles in NMSC prediction. Nonetheless, our goal was to determine how an ANN would perform using data most commonly available in the EMR, which typically does not include UVR or NMSC family history information. We expect that if the EMR consistently recorded personal UVR exposure and NMSC family history and our same algorithm was extended to include this information, our results would substantially improve.

Another analysis endeavored to create a cancer prediction rule using logistic regression and clinically relevant parameters^26^. They used 481 patients with cancer and 481 controls, with parameters that included UVR exposure, exercise, prior skin cancer history, and family history of skin cancer. The study yielded 40% sensitivity and 87% specificity for BCC and 39% sensitivity and 92% specificity for SCC, with sensitivities half of what our model produces and specificity 20–30% better.

While it is clear that our model would benefit greatly from the inclusion of personal UVR exposure and family history of skin cancer information, we believe that the fact that our model performed well without these criteria is noteworthy. Additional parameters that would also be expected to improve our model would be history of organ transplantation and concomitant immunosuppression (to avoid rejection), industrial carcinogen exposures (e.g. mutagenic polycyclic aromatic hydrocarbons), and clinical features of MC1R deficiency (e.g. red hair, freckles). A single-center prospective study of Queensland renal transplant patients revealed that the overall NMSC incidence was 28.1% and increased with each extension of the immunosuppressive treatment^27^.

Recently a single convolutional neural network has been used to classify skin cancer and achieved performance on par with 21 board-certified dermatologists across three critical diagnostic tasks: keratinocyte carcinoma classification, melanoma classification and melanoma classification using dermoscopy^10^. While this neural network was primarily designed to aid skin cancer diagnosis based on clinical images, our neural network is used for prediction of non-melanoma skin cancer risk based on personal health informatics. Both studies reveal the potential for neural network approaches to improve the diagnosis and/or management of patients at risk of NMSC.

Moving forward, we envision that the developed ANN could help direct primary care physicians in decision making on which patients are at highest risk for skin cancer, with subsequent referral to dermatology for total body skin examination. With readily available personal health data, this model can be easily implemented in a mobile app, on a website, or even integrated into an EMR system. This would allow the clinicians to access the information and patient risk status immediately when the data is entered, hence detecting and preventing cancer at its early stage.

## Conclusion

We have developed a multi-parameterized artificial neural network that is able to predict and stratify an individual’s risk of developing non-melanoma skin cancer (NMSC) based solely on select personal health informatics. Our approach is easy-to-implement, non-invasive, and cost-effective while achieving comparable sensitivity and specificity to other approaches which often require ultraviolet radiation exposure and family history data. While it is anticipated that our model would likely be further improved with such information included, more clinical testing is needed and on the way.
